# What Happens to Cognition and the Daily Use of Technology in Older Adults? Working Memory as a Key Cognitive Function

**DOI:** 10.1002/alz70858_103004

**Published:** 2025-12-25

**Authors:** Estela Benitez, Maria Celeste López Moreno, Renata Diaz Colodrero, Manuel Obando Ulloa, Guillermina Quenan, Melania Lope, Carla Perez, Leticia Vivas, María Martha Richard's, Daiana Bario

**Affiliations:** ^1^ Faculty of Health Sciences and Social Work of the National University of Mar del Plata (UNMDP), Mar del Plata, Buenos Aires, Argentina; ^2^ National Council of Scientific and Technical Research (CONICET), Mar del Plata, Buenos Aires, Argentina; ^3^ Institute of Basic and Applied Psychology and Technology (IPSIBAT), National University of Mar del Plata (UNMDP), Psychology Faculty, Mar del Plata, Buenos Aires, Argentina

## Abstract

**Background:**

This study investigates the association between the frequency of daily use of technology and working memory (WM) cognitive function in older adults, considering variables such as digital competencies, frequency of technology use, educational level, and age. Previous research shows mixed results; some indicate benefits (Pochintesta et al., 2021; Valdivieso Ortega et al., 2021), while others find inconclusive evidence (Montejo et al., 2022). Declining WM, commonly associated with aging (Stern, 2021) and significantly affected by neurodegenerative diseases such as Alzheimer's (Calero & Navarro, 2022), manifests as a reduced capacity to process and store information, leading to difficulties in daily life. This research used a reverse sentence WM test, increasing in difficulty, based on Baddeley's model, which defines WM as retaining and manipulating verbal information via the phonological loop, central executive, and episodic buffer. (Baddeley & Hitch, 1974; Baddeley, 2000).

**Method:**

The sample consisted of 66 individuals aged over 60, predominantly women (90.9%), from the University Program for Older Learners (PUAM) at the National University of Mar del Plata and community centers of Mar del Plata city. The average age was 69.4 years, with an average educational level of 5 (tertiary or incomplete universitary education) (Pascual et al., 1993). Participants completed a reverse sentence WM test, the Digital Competencies Questionnaire (DigComp, 2022, IKANOS), and a frequency test for everyday technology use.

**Result:**

A Kendall´s Tau correlation model was applied due to data non‐normality (Shapiro‐Wilk test, *p* < 0.05). Significant positive correlations were found between WM and digital competencies (*p* < .001), frequency of technology use (*p* < .01), and educational level (*p* < 0.01), and showed a negative significant correlation with age (*p* < .05).

**Conclusion:**

Findings suggest that older adults with higher WM performance also have higher educational levels, supporting education as a protective factor in aging. Higher WM scores were associated with greater digital competencies and more frequent use of everyday technology, contributing to independence and autonomy. However, despite this, older participants showed lower WM scores, consistent with age‐related cognitive decline. These results highlight the importance of promoting technology access among older adults to enhance digital skills and maintain cognitive function.

